# AtKinesin-13A is located on Golgi-associated vesicle and involved in vesicle formation/budding in Arabidopsis root-cap peripheral cells

**DOI:** 10.1186/1471-2229-9-138

**Published:** 2009-11-25

**Authors:** Liqin Wei, Wei Zhang, Zhaohui Liu, Yan Li

**Affiliations:** 1 State Key Laboratory of Plant Physiology and Biochemistry, College of Biological Sciences, China Agricultural University, Beijing 100193, PR China; 2 Research Center of Molecular and Developmental Biology, Key Laboratory of Photosynthesis and Environmental Molecular Physiology, Institute of Botany, Chinese Academy of Sciences, Beijing 100093, PR China

## Abstract

**Background:**

AtKinesin-13A is an internal-motor kinesin from Arabidopsis (*Arabidopsis thaliana*). Previous immunofluorescent results showed that AtKinesin-13A localized to Golgi stacks in plant cells. However, its precise localization and biological function in Golgi apparatus is unclear.

**Results:**

In this paper, immunofluorescent labeling and confocal microscopic observation revealed that AtKinesin-13A was co-localized with Golgi stacks in Arabidopsis root tip cells. Immuno-electron microscopic observations indicated that AtKinesin-13A is primarily localized on Golgi-associated vesicles in Arabidopsis root-cap cells. By T-DNA insertion, the inactivation of the *AtKinesin-13A *gene (NM-112536) resulted in a sharp decrease of size and number of Golgi vesicles in root-cap peripheral cells. At the same time, these cells were vacuolated in comparison to the corresponding cells of the wild type.

**Conclusion:**

These results suggest that AtKinesin-13A decorates Golgi-associated vesicles and may be involved in regulating the formation of Golgi vesicles in the root-cap peripheral cells in Arabidopsis.

## Background

Kinesins are a large super-family of microtubule motor proteins that can use the energy of ATP hydrolysis to produce force and move along microtubules [1,2]. Based on their motor domain location within the primary sequence of the proteins, different kinesins may have their motor domains affixed at C-terminal, N-terminal or internal positions [3]. The C-terminal and N-terminal motor kinesins transport various vesicles and organelles toward the microtubules minus-terminal or plus-terminal, respectively. The internal motor kinesins found in animal cells are not able to move along the microtubules in the conventional form, but instead depolymerize microtubules from both ends [4]. The completed Arabidopsis genome contains at least 61 genes encoding polypeptides with the kinesin catalytic core. Among these kinesins, AtKinesin-13A and AtKinesin-13B are two internal-motor kinesins [5,6]. However, the similarity of AtKinesin-13A and AtKinesin-13B to kinesins of the same subfamily from other kingdoms is only limited to the catalytic core, and they lacks a Lys-rich neck motif commonly found in animal Kinesin-13s. Plant Kinesin-13A and animal Kinesin-13s also have different localization patterns [7,8]. Lu et al. reported that AtKinesin-13A was co-localized with Golgi stacks in various Arabidopsis cells, indicating that AtKinesin-13A is a special plant internal-motor kinesin [8]. However, the precise localization of AtKinesin-13A as well as its biological function in plant Golgi apparatus is unclear.

Cellular trafficking is the foundation of cellular morphology and function, where the Golgi apparatus plays an important role in the secretion and transportation of cellular vesicles [9]. In animal cells, the Golgi apparatus is positioned near the microtubule-organizing center, and its localization and organization depend on intact microtubules [10]. In addition, microtubules and microtubule-based motor proteins play critical roles in Golgi dynamics [11,12]. Both the conventional kinesins and kinesin-related proteins have been reported to regulate Golgi structure and function in animal cells [13-19]. Actin microfilaments have also been found to be necessary in maintaining the sub-cellular localization of the animal Golgi complex [20]. Both microtubules and microfilaments cooperate in maintaining the balance of Golgi dynamics within animal cells.

Unlike in animal cells, the Golgi apparatus of plant cells consists of a large number of small, independent Golgi stacks that are distributed throughout the cytoplasm [21-23], with the number of Golgi stacks being different among different kind of cells. The number of the Golgi apparatus is typically abundant in plant root-cap peripheral cells, in which very large vesicles are produced by each Golgi apparatus [24]. This is in accord with the high secretory activity needed for root growth in soil [25]. On the other hand, it is generally believed that the movement of plant Golgi stacks is solely dependent on actin microfilaments [23].

In plant cells, it has been reported that microtubules play a key role in organelle movement [26-29], but it is unclear whether microtubule-based motor kinesins take part in regulating the structure and function of Golgi apparatus. In the present study, AtKinesin-13A was detected on Golgi-associated vesicles in root-cap cells of Arabidopsis. Additionally, the Golgi structure was abnormal in root-cap peripheral cells of the *kinesin-13a-1 *mutant. These results suggest that AtKinesin-13A may participate in regulating the formation of Golgi-associated vesicles in Arabidopsis root cap peripheral cells.

## Results

### AtKinesin-13A co-localization with Golgi stacks in Arabidopsis root tip cells

**Figure 1:**
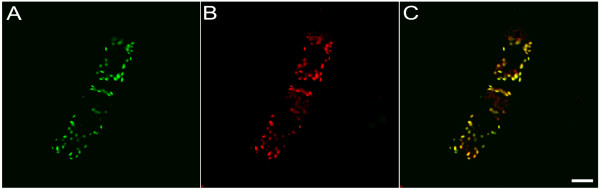
**Immuno-localization and confocal microscopy observation showed co-localization of AtKinesin-13A and the Golgi stacks in Arabidopsis root tip cells**. (A) Nag-GFP showed the distribution of Golgi stacks in Arabidopsis root tip cells. (B) Immunofluorescence labeling showed the distribution of AtKinesin-13A in Arabidopsis root tip cells. (C) A merged image had AtKinesin-13A signal pseudo-colored in red and Nag-GFP in green, showing co-localization of AtKinesin-13A and the Golgi apparatus in Arabidopsis root tip cells. Bar: 5 μm.

The expression of AtKinesin-13A was not tissue-specific in *Arabidopsis *[30]. On the other hand, there are different types of Golgi stacks in plant root tip cells. Therefore, for further studying the localization and function of AtKinesin-13A, Arabidopsis root tip cells were used. *N*-acetylglucosaminyl transferase I (Nag)-GFP fusion protein specially decorates Golgi stacks in plant cells [8]. To co-localize AtKinesin-13A with Golgi apparatus in Arabidopsis root tip cells, we used an Arabidopsis line expressing the Nag-GFP fusion. Root tip cells were used to verify the relationship between AtKinesin-13A localization and individual Golgi stacks marked by Nag-GFP. Confocal microscopic observation revealed that AtKinesin-13A was co-localized with Nag-GFP in Arabidopsis root tip cells (Fig. 1), suggesting that AtKinesin-13A is localized to the Golgi stacks in these cells.

### AtKinesin-13A is mainly localized on Golgi vesicles in Arabidopsis root-cap cells

**Figure 2:**
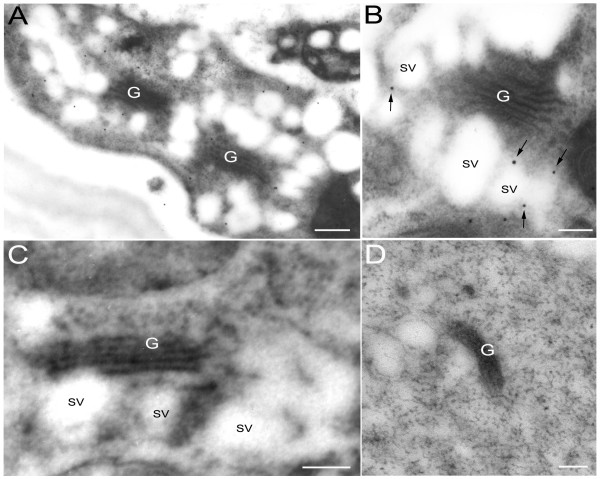
**Immuno-gold labeling and electron microscopic observation showed that AtKinesin-13A was located on Golgi-associated vesicle in root cap cells of Arabidopsis**. AtKinesin-13A was labeled with the purified AtKinesin-13A antibody. The AtKinesin-13A antibody was detected with a secondary antibody with 10 nm gold particles. (A) Electron microscopic observation showed that AtKinesin-13A was located mainly on Golgi-associated vesicle in root cap cells of Arabidopsis. (B) Note the labeling on the margin of Golgi vesicles in Arabidopsis root cap cells (arrows). (C) Control section, incubated with the secondary antibody alone, did not show gold particles association with Golgi vesicles. (D) Atkinesin-13A antibody could not label Golgi vesicles in the section of root cap cells in *kinesin-13a-1 *mutant line. G: Golgi apparatus. SV: secretory vesicles. Bars: 200 nm (A, D); 150 nm (B, C).

To determine the localization of AtKinesin-13A on Golgi stacks at the ultra-structural level, ultra-thin sections were immuno-gold labeled with anti-AtKinesin-13A antibody in Arabidopsis root-cap cells. The immuno-gold labeling with the affinity-purified anti-AtKinesin-13A antibody and electron microscopy revealed that AtKinesin-13A was specifically linked with the Golgi stacks of Arabidopsis root cap cells. Electron microscopic observation detected that gold particles were associated with the Golgi vesicles in the root-cap cells (Fig. 2A). Quantitative analysis of the gold particles distribution showed a preferential association of AtKinesin-13A with the Golgi vesicle, accounting for 55.6% of the total gold particles (Table 1). We additionally found that gold labeling was located mainly on the margin of Golgi vesicles in Arabidopsis root cap cells (Fig. 2B, arrows). This result suggests that AtKinesin-13A may locate on membranes of Golgi vesicles in these cells. Control sections, incubated with the secondary antibody alone, did not show gold particles association with Golgi vesicles (Fig. 2C). In addition, we also found that Atkinesin-13A antibody can not label Golgi vesicles in the root cap cells of *kinesin-13a-1 *mutant line (Fig. 2D).

**Table 1:** Sub-cellular distribution of AtKinesin-13A in root-cap cells of Arabidopsis (mean ± SD) (N = 15).

Golgi-associated vesicles (%)	Other vesicles (%)	Non-vesicles (%)
55.6 ± 1.6	20 ± 1.2	24.4 ± 2.1

Note: number represented the percentages (mean ± SD) of the total labeling in distinct locations in root-cap cells of Arabidopsis.

N: the number of cells analyzed. Golgi-associated vesicles: the vesicles around Golgi stacks. Other vesicles: the vesicles beyond Golgi stacks. Non-vesicles: cytoplasmic labeling not associated with vesicles or Golgi vesicles.

### *AtKinesin-13A *gene inactivation caused obvious structural changes of Golgi stacks in root cap peripheral cells

Lu et al [8] reported two independent Arabidopsis T-DNA insertion mutations at the *AtKinesin-13A *locus, which led to the loss of function of Kinesin-13A in Arabidopsis. In Lu et al. paper, it was concluded that two Atkinesin-13A mutant lines (*kinesin-13a-1 *and *kinesin-13a-2*) exhibited identical phenotypes. They have confirmed that the mutant phenotypes were indeed caused by the T-DNA insertion at the Kinesin-13A locus based on their complementation results [8]. The *kinesin-13a-1 *mutant line was used for current study.

**Figure 3:**
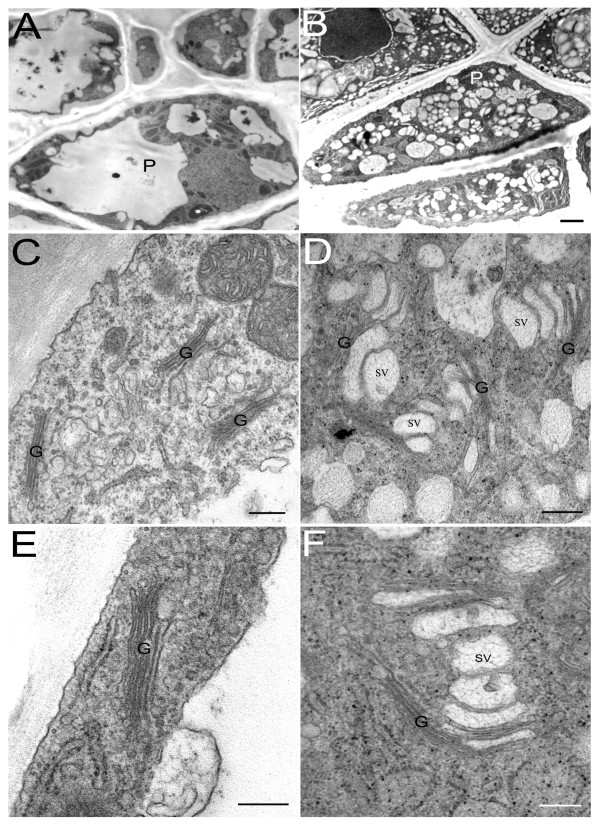
**Electron microscopic observation showed obvious structural changes of Golgi apparatus in root cap peripheral cells of the *kinesin-13a-1 *mutant line**. (A) The peripheral cells of the *kinesin-13a-1 *mutant lines contained a few large vacuoles and few vesicles. (B) The peripheral cells of the wild type root cap contained numerous vesicles. (C), (E) Golgi-associated vesicles were rare and small in the peripheral cells of the *kinesin-13a-1 *mutant. (D), (F) The wild type root-cap peripheral cells contained abundant and bulky secretory vesicles around the Golgi stack. G: Golgi apparatus. P: peripheral cells. SV: secretory vesicles. Bars: 1 μm (A, B); 200 nm (C, D); 150 nm (E, F).

The Golgi apparatus is the main executer of secretory activity in root-cap peripheral cells [24]. Root-cap peripheral cells of the *kinesin-13a-1 *mutant were compared with those of wild-type Arabidopsis using transmission electron microscopy. Peripheral cells of the *kinesin-13a-1 *mutant lines contained a few large vacuoles, but few vesicles (Fig. 3A). In contrast, numerous vesicles were found in the peripheral cells of the wild type root cap (Fig. 3B). In addition, Golgi-associated vesicles were also rare and small in the peripheral cells of the *kinesin-13a-1 *mutant (Fig. 3C, E), compared to how abundant secretory vesicles around the Golgi stack in wild type root-cap peripheral cells (Fig. 3D, F). A different morphology was also found in cisternal morphology of Golgi stacks between wild and mutant line. Normally, cisternae swell at the ends in Golgi stacks of root-cap peripheral cells (Fig. 3D). However, this does not occur in the *kinesin-13a-1 *mutant line (Fig. 3C). Therefore, it appeared that the morphology of the Golgi apparatus in the *kinesin-13a-1 *mutant line is significantly different from that of the wild type for root-cap peripheral cells.

In the meristematic cells and columella cells of the root cap, however, the Golgi morphology of the *kinesin-13a-1 *mutant was not significantly different from that of wild type (data no shown).

## Discussion

Golgi apparatus is a vital organelle in the process of cellular secretion. In animal cells, the high level of activity at the Golgi apparatus is sustained largely through the combined effects of microtubules, actin-microfilaments, and some intermediate filaments [31]. In plant cells, the Golgi apparatus consists of a large number of small, independent Golgi stacks that appear to be randomly distributed throughout the cytoplasm that take on rapid stop-and-go movements [21,22,32]. The Golgi apparatus is a polar organelle. From its *cis*-cisternae to the *trans*-network, there are multi-compartments that carry out versatile functions. Within different functional compartments there are also special proteins that perform different biological functions. Previous studies have shown that a number of molecular motors are around Golgi apparatus and involved in maintaining its proper structure and function in animal cells [31]. However, few motors were found to locate on plant Golgi apparatus before. Recently, Lu et al reported that AtKinesin-13A decorated Golgi stacks of various Arabidopsis cells [8]. Results from immuno-gold labeling and electron microscopy presented here further indicated that AtKinesin-13A located to the margin of Golgi vesicles in Arabidopsis root cap cells. This result suggests that AtKinesin-13A may associate with membranes of Golgi vesicles in Arabidopsis root cap cells. On the other hand, there is no predicted trans-membrane domain in the Atkinesin-13A protein sequence. Taken together, these results imply that AtKinesin-13A may be a cytoplasmic oriented peripheral membrane protein of Golgi-associated vesicles in Arabidopsis.

The root cap consists of a large number of parenchyma cells. During the growth of the root system, root-cap cells initially stem from the root-cap meristem by mitosis, then progress through a series of distinctive developmental stages. Ultimately, these cells separate from the periphery of the root cap to produce border cells [33]. During differentiation from meristematic cells into peripheral cells, the number of Golgi stacks per cell as well as the size and the number of Golgi-associated vesicles per Golgi apparatus increase visibly. In root-cap peripheral cells, there are large populations of active secretory Golgi apparatus, and the secretory vesicles around the Golgi are large and abundant, while the size and number of Golgi-associated vesicles in root-cap meristematic cells are relatively few and small [24]. In this paper, electron microscopy observation showed that the *AtKinesin-13A *gene inactivation induced a significant decrease of the size and number of Golgi-associated vesicles in root-cap peripheral cells. In addition, no swelling was observed at the ends of *trans*-cisternae of Golgi stacks in root-cap peripheral cells of the mutant line. The large Golgi-associated vesicles often come from *trans*-cisternae swelling and budding in root-cap peripheral cells [22,34]. These results suggest that AtKinesin-13A may be involved in the *trans*-cisternae swelling and budding of Golgi-associated vesicles in root-cap peripheral cells, and then regulates the size and number of Golgi-associated vesicles in these cells.

The expression of AtKinesin-13A was not tissue-specific in Arabidopsis [30]. However, some unconventional Golgi apparatus behaviors were only observed in the root-cap peripheral cells of the Arabidopsis *kinesin-13a-1 *mutant line. Recently, Poulsen et al also reported that Aminophospholipid ATPase3 (ALA3), a member of the P4-ATPase subfamily in Arabidopsis, localizes to the Golgi stacks and that mutations of ALA3 result in devoid of the characteristic Golgi vesicles in only Arabidopsis root-cap peripheral cells [35]. Taken together, these results indicated that both AtKinesin-13A and ALA3 mutations have similar phenotype of Golgi vesicles in root-cap peripheral cells. The root-cap peripheral cells secrete mucilage to protect and lubricate root cap as they force their way between soil particles. Hence the Golgi apparatus in root-cap peripheral cells are very specialized and possess a high secretory activity. So there may be some special Golgi-associated vesicles or some special vesicle formation/budding mechanism in root-cap peripheral cells in which the Atkinesin-13A and ALA3 play essential roles.

## Conclusion

In this paper we found that AtKinesin-13A located on Golgi-associated vesicles in Arabidopsis root-cap cells, and the inactivation of the *AtKinesin-13A *gene caused a sharp decrease of Golgi vesicles number and size in root-cap peripheral cells. Based on these results, we speculate that there may be a novel mechanism by which AtKinesin-13A controls Golgi vesicles formation or budding in plant root cap peripheral cells.

## Methods

### Plant materials

The *Arabidopsis thaliana *plants used were the ecotype Columbia. The Arabidopsis *kinesin-13a-1 *mutant line and the Arabidopsis line expressing *N*-acetylglucosaminyl transferase I (Nag)-GFP used in our experiments were described in Lu et al [8]. Arabidopsis seeds were germinated on solid medium containing MS salt and 0.8% agar under long day conditions (16 h of light/8 h of dark, 20°C) in Petri dish plates. The 5- to 6-day seedlings of the Arabidopsis were used for the experiments.

### Immunofluorescence labeling

The fixative procedure was similar to that in our previous report [36]. The seedlings of the Arabidopsis line expressing Nag-GFP were fixed for 1 hour in freshly prepared 4% paraformaldehyde in 50 mM Pipes (pH6.9). Following three washes in 50 mM Pipes buffer, the samples were incubated in an enzyme solution containing 1% cellulase and 1% pectinase (50 mM Pipes buffer containing 40 μM phenylmethylsulfonyl fluoride (PMSF) to inhibit the protease activity) at room temperature for 8 min. After further three washes with 50 mM Pipes buffer, the release procedure of root tip cells was conducted according to Liu et al [37].

The immunofluorescence labeling of slides containing Arabidopsis root tip cells was processed as described by Lee and Liu [38] with slight modifications. In brief, the cell was incubated in 1% Triton X-100 in PBS for 1 hour at room temperature, followed by a rinse in PBS. The cells were then treated with the purified AtKinesin-13A antibody (diluted at 1:60 in PBS) overnight at room temperature. The previous report has indicated that the purified AtKinesin-13A antibody could label specific AtKinesin-13A protein in Arabidopsis cells [8]. After a further washing, the secondary goat anti-rabbit TRITC-conjugated antibody (Sigma Company, diluted 1:100 in PBS) was added and allowed to react for 1.5 hours at 37°C. In the control treatment, the primary antibody was omitted. In that case, no staining was detected.

### Immuno-gold labeling and electron microscopic observation

Arabidopsis root tips were processed for immuno-gold labeling as described by Van den Bosch and Newcomb [39], and modified as Chen et al [40]. In brief, Arabidopsis root tips were fixed and dehydrated. Then the materials were embedded in L R White acrylic resin (Sigma Company). Polymerization of L R White was brought about by heat-curing the resin at 46°C for 16 hours.

The sections were then placed in 3% (v/v) fish gelatin (Sigma) in a PBS buffer for 1 hour, followed by primary antibody incubation for 1 hour at room temperature. Then after rinsing in PBS, secondary antibody was added and incubated for 1 hour at room temperature. The sections were then rinsed in PBS. The purified rabbit anti-AtKinesin-13A antibody diluted 1:60 in PBS containing 3% (v/v) fish gelatin (Sigma) served as the primary antibody [8]. The secondary antibody was a goat anti-rabbit antibody conjugated with 10-nm colloidal gold particles (Sigma Company, diluted 1:60 in PBS containing 3% fish gelatin). For the controls, the primary antibody was omitted, or the root tip cells of *kinesin-13a-1 *mutant line were labeled. The samples were observed and photographed under a JEM-100S or JEM 1230 electron microscope at 80 kV.

To estimate specificity of labeling, quantitative evaluations were carried out on ultra-thin sections. The gold particles were counted and ascribed to one of the following categories: Golgi-associated vesicles, vesicles, or non-vesicles. The numbers in table 1 represented the percentage (mean ± SD) of the total labeling in distinct locations in whole cytoplasm.

### Conventional transmission electron microscopic observation

The Arabidopsis seedlings of wild and *kinesin-13a-1 *mutant line were harvested and Arabidopsis root tips were fixed in 2.5% glutaraldehyde in 50 mM Pipes buffer, pH 6.8, for 1 hour at room temperature. Specimens were washed in the Pipes buffer and post-fixed for 2 hours in 1% osmium tetroxide. Arabidopsis root tips were then dehydrated in an acetone series and embedded in Spurr's resin (SPI Supplies). Polymerization of the resin was conducted by heat-curing the resin at 70°C for 18 hours. Thin sections were then collected on formvar-coated gold grids.

The peripheral, columella, and root-cap meristematic cells were observed. Both wild-type and *kinesin-13a-1 *mutant line were processed and observed in the same condition. Sections were stained with 2% uranyl acetate for 10 min and 1% lead citrate for 20 min before being observed and photographed at 80 kV with a JEM-100S or JEM-1230 electron microscope.

## Authors' contributions

LW carried out the immuno-labeling and microscopy observation, and drafted the manuscript. WZ carried out the conventional transmission electron microscope observation. ZL participated in the conventional transmission electron microscope observation. YL conceived of the study, and participated in its design and coordination and helped to draft the manuscript. All authors read and approved the final manuscript.
